# Mechanism of the influence of basalt fiber parameters on the mechanical properties and microstructure of high-volume red mud-cement paste

**DOI:** 10.1038/s41598-026-47848-1

**Published:** 2026-04-10

**Authors:** Peipei Li, Tao Xie, Mingxuan Shen, Jinghang Xia, Jie Tang

**Affiliations:** Department of Civil Engineering, Guizhou University, Guiyang, 550025 Guizhou China

**Keywords:** Red mud, Basalt fiber, Cement paste, Mechanical properties, Microstructure, Engineering, Materials science

## Abstract

Red mud (RM) is a highly alkaline solid waste generated by the alumina industry. The incorporation of high-volume red mud into cement-based materials often leads to mechanical degradation and microstructural instability, highlighting an urgent need for verifiable enhancement pathways and mechanistic explanations. Based on the established red mud-cement paste system, this study systematically evaluates the effects of red mud content on the mechanical properties and microstructure of the cement-based materials. Furthermore, a full-factorial design considering the “length × dosage” parameters of basalt fibers (BF) is implemented to investigate the evolution patterns of compressive and flexural strengths at 3, 7, and 28 days, as well as their correlations with structural characteristics. The results indicate that without fiber reinforcement, 30% red mud content reduces the 28-day compressive strength from 55.5 MPa to 40.2 MPa, with a slight decrease in flexural strength. Upon fiber incorporation, the effects exhibit phased evolution: At 3 days, fiber length and dosage jointly drive compressive strength improvement while having limited impact on flexural strength; At 7 days, synergistic interaction between length and dosage emerges, showing combination-dependent effects; At 28 days, fiber length dominates compressive strength development while dosage primarily governs flexural strength. Among the investigated fiber-reinforced 30% RM mixtures, the combination of 12 mm fiber length and 0.1% fiber dosage provided the best overall balance of mechanical performance. Although its 28-day compressive strength of 38.95 MPa was slightly lower than that of the fiber-free R30 specimen of 40.20 MPa, its flexural strength increased by 0.6 MPa, and the reinforced specimen exhibited fewer macroscopic cracks and better structural integrity after failure. The microscopic analysis reveals that the incorporation of red mud shifts the hydration products from predominantly crystalline phases to amorphous and layered structures. The fibers contribute through bridging and pull-out energy dissipation, while their interfacial interlocking mechanism suppresses continuous crack propagation and enables performance compensation. This study establishes a replicable evaluation framework and provides operable proportioning windows for parameter optimization and mechanistic analysis in high-volume red mud-cement paste systems.

**Supplementary Information:**

The online version contains supplementary material available at 10.1038/s41598-026-47848-1.

## Introduction

 China is the world’s largest producer of alumina. As of the first half of 2025, its alumina production capacity has reached 111 million tons per year. Red mud is a bulk industrial waste generated during the refining of alumina from bauxite^1,2^. It is estimated that producing 1 ton of alumina yields 1–2 tons of red mud^3,4^. In 2024, China’s annual red mud output reached 115 million tons, with a cumulative stockpile of 1.5 billion tons. The utilization volume stood at 13 million tons, resulting in a utilization rate of less than 12%^5^.The low utilization rate of red mud is primarily constrained by technical bottlenecks and insufficient large-scale application. Its strongly alkaline nature exerts significant pressure on the environment and land resources^6–10^. Therefore, achieving high-efficiency resource utilization of red mud in the construction materials sector has become a critical issue requiring urgent resolution.

Cement (P·O), as a vital construction material, is a potential source of environmental pollution due to significant CO₂ emissions generated during its production^11–13^. Each ton of cement produced releases 510 to 712 kg of CO₂ into the environment, primarily resulting from the chemical process of high-temperature limestone calcination^14^. This accounts for approximately 7% of global atmospheric CO₂ emissions^15^.Therefore, there is an urgent need to minimize cement consumption in the construction industry. Red mud contains abundant active components such as SiO₂, Al₂O₃, and CaO, which are similar to cement constituents. Simultaneously, the amorphous aluminosilicate substances present in red mud can undergo a secondary pozzolanic reaction with calcium hydroxide released during cement hydration^16,17^, thereby developing cementitious properties. Wu et al.^1^ proposed strategies to promote the extensive utilization of red mud for carbon reduction, thereby providing a theoretical framework and technical support for the green transformation of the aluminum industry. Ansari et al.^18^ investigated the potential use of red mud, which is produced at approximately 1.5 tons per ton of primary alumina produced, as supplementary cementitious materials (SCMs) in sustainable construction. They also analyzed its chemical and mineralogical properties, pozzolanic activity, and associated environmental and health risks to assess the feasibility of utilizing red mud (RM) as supplementary cementitious materials. Consequently, red mud can partially replace cement as a binding material^19,20^. In recent years, researchers have focused on investigating the effects of RM on the mechanical properties, durability, and microstructure of cement-based materials^4,21–26^. Ghavan et al.^21^ partially replaced cement with red mud at varying proportions (0%- 20%), ultimately finding that 12% red mud yielded the maximum strength and favorable microstructure. D et al.^22^ demonstrated that red mud can replace up to 15% of cement, beyond which compressive strength and splitting tensile strength begin to decline. Their study confirmed that the maximum replacement ratio of cement with red mud should not exceed 15%. Luo et al.^4^ found that cement paste incorporating 15% red mud calcined at 1000 °C exhibited significantly higher compressive strength than pure cement paste, while the strength of paste containing 30% calcined red mud was essentially equivalent to that of pure cement paste. Vladić Kancir et al.^23^ investigated the synergistic effects between RM and common supplementary cementitious materials in cement systems with 20% red mud content, demonstrating the feasibility of achieving higher cement replacement levels. Salim et al.^24^ experimentally demonstrated that replacing cement with red mud within the 10%- 20% range significantly enhances the mechanical properties and durability of cement-based composites. Yan et al.^25^ revealed that substituting 20% ordinary Portland cement (OPC) with RM is crucial for producing curable ultra-high performance concrete mixtures. However, when the RM content exceeds 30%, it increases porosity and water absorption, with the 28-day compressive strength experiencing a maximum reduction of 32.9%. Hou et al.^26^ observed a significant decrease in the compressive strength of ultra-high performance concrete when the RM replacement level reached 40%. These studies demonstrate that without mechanical activation^27^ or thermal treatment^28,29^, red mud replacement below 20% has minimal or even beneficial effects on performance. Tan^30^ reported that when the red mud content was 20%, the 5 h and 1 d compressive strengths of the “red mud–cement” cementitious material decreased by 30.68% and 38.46%, respectively, compared to those of the cement paste. Therefore, to increase the cement replacement ratio with red mud, it is necessary to incorporate fibers or other materials to enhance the performance of red mud-cement based materials.

Fiber reinforcement technology serves as an effective method for enhancing the mechanical properties of cement-based materials. The incorporation of fibers can inhibit the initiation and propagation of micro-cracks within the material^31^, thereby significantly improving the strength and toughness of the hardened system^32–34^. Among various fiber materials, BF is regarded as a green high-performance fiber due to its high tensile strength, high elastic modulus, and excellent alkali and corrosion resistance^35,36^. BF demonstrates more stable long-term strain performance compared to other fibers^37^.Basalt fiber exhibits comparable coefficients of expansion and contraction to cement^38^. When mixed with cement-based materials, it demonstrates natural compatibility, ease of dispersion, and favorable chemical stability. Applying the fiber reinforcement strategy to red mud-cement binder systems is expected to partially counteract the increased porosity and strength reduction induced by high-volume red mud, thereby enhancing material performance and expanding the application scope of red mud. Although previous research have explored the reinforcing effects of polyvinyl alcohol fibers^39,40^ or steel fibers^41,42^ in concrete, the mechanisms underlying the performance of basalt fiber in cementitious systems remain insufficiently investigated. Song et al.^43^ demonstrated that basalt fiber significantly contributes to the early-age compressive and flexural strength of basalt fiber sprayed comentitious composites (BFSCC). Chakkor et al.^44^ investigated the effects of basalt fiber at varying contents (0.4%, 0.8%, and 1.2% by mass) on mortar durability and strength, revealing that the 1.2% dosage substantially enhances the performance of geopolymer mortar. Zhang et al.^45^ emphasized the critical role of harmful pores in determining the compressive strength of basalt fiber-reinforced coral sand concrete (BFRCSC), identifying that the BF-0.1 mixture with 0.1% basalt fiber content achieved the highest strength. Wang et al.^46^ conducted experimental investigations on the workability and other properties of red mud-based grouts reinforced with polypropylene, glass, and basalt fibers. The developed grout mixtures improved the utilization of red mud in geotechnical engineering applications. Qin et al.^47^ examined the effects of new basalt fiber (NBF) content and length on the workability and mechanical performance of concrete, identifying optimal fiber parameters as 1.5–2.0 kg/m³ content and 18–24 mm length. However, current research on fiber reinforcement in red mud cementitious materials remains limited. Xu et al.^48^ concluded that appropriate amounts of steel fibers and nano-SiO₂ can enhance the performance of red mud-based geopolymer concrete, with their hybrid use yielding the most favorable results. Nazir et al.^49^ developed fiber-reinforced metakaolin-red mud geopolymer mortar, demonstrating that fiber incorporation improves the strength characteristics of geopolymer mortar. Liu et al.^50^ developed basalt fiber-reinforced red mud concrete and found that fiber dosage has a more pronounced effect on strength improvement compared to fiber length, with the optimal dosage identified as 0.2%. In another study, Liu et al.^51^ formulated red mud concrete by replacing 40% of cement with red mud, revealing that specimens with hybrid fibers (when the polyvinyl alcohol fiber content was 0.15% and the basalt fiber content was 0.1%, when the polyvinyl alcohol fiber content was 0.15% and the basalt fiber content was 0.2%) achieved the optimal 28-day compressive and flexural strengths.

Based on this background, the present study employs a red mud-cement composite as the binding matrix and incorporates BF with four lengths (6 mm, 9 mm, 12 mm, and 15 mm) and three volume fractions (0.1%, 0.3%, and 0.5%) to prepare fiber-reinforced red mud-cement paste specimens. Experimental investigations were conducted on their mechanical properties. By testing the compressive and flexural strengths of paste specimens at 3, 7, and 28 days, this research systematically evaluates the effects of BF length and volume fraction on the mechanical performance of red mud-cement paste, revealing the evolution patterns of fiber reinforcement effects over time. Based on the experimental results, the optimal BF length and content were determined when the RM substitution rate for cement reached 30%. In addition, the mechanisms behind the improvement in mechanical properties were elucidated using X-ray diffraction (XRD), scanning electron microscopy (SEM), and thermogravimetric analysis (TG). Since this study adopts a cement paste system, the experimental design eliminates interference from aggregate factors, thereby more prominently highlighting the direct interaction mechanisms between fibers and the cementitious matrix. The findings provide valuable experimental evidence and theoretical support for the efficient utilization of red mud in cementitious materials and further investigation into fiber reinforcement mechanisms.

## Experimental design

### Raw materials

The RM used in this study was obtained from Guanglv Alumina Co., Ltd. in Guizhou, China. As RM is an industrial solid waste stored in open-air stockpiles and mainly exists in block form, it was first ground using a ball mill. The ground material was then sieved through a 30-mesh (0.6 mm) electric vibrating screen. The fraction with particle sizes smaller than 0.6 mm was subsequently dried in an oven at 105 °C for 4.5 h to produce an active RM powder.

**Fig. 1:**
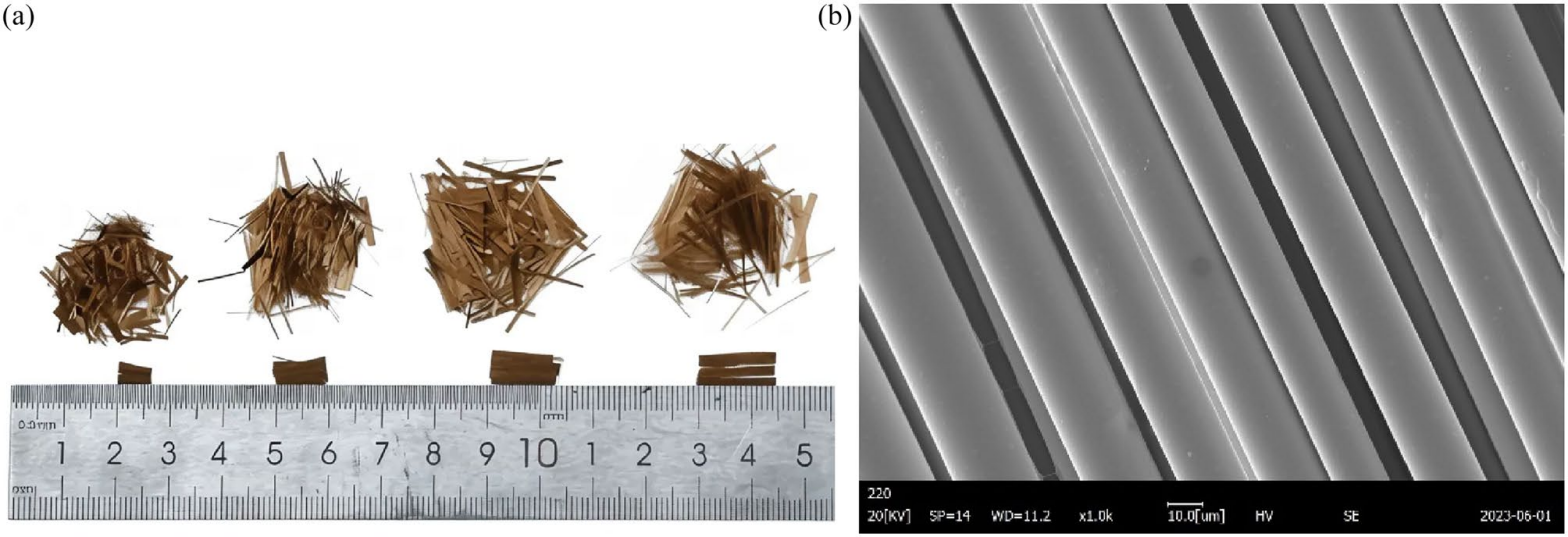
Macroscopic and microscopic morphology of BF. (**a**) Macroscopic morphology; (**b**) Microscopic morphology.

The cement used in this experiment was Conch-brand 42.5 ordinary Portland cement produced in Guizhou Province. The BF employed in this study was supplied by a composite materials company in Jiangsu Province, China. The macro- and micro-morphologies of BF are shown in Fig. 1. The physical properties of BF were shown in Table 1. The mixing water used in this experiment was ordinary tap water from Guiyang City, which meets the requirements specified in the standard JGJ63-2006. The main chemical compositions of the raw materials, determined by X-ray fluorescence (XRF), are listed in Table 2.

**Table 1.** Physical and mechanical properities of BF.

Density(g·cm^− 3^)	Diameter(µm)	Tensile strength(MPa)	Elastic modulus(GPa)	Fracture elongation(%)
2.65	17	1838	78	2.1

**Table 2.** Chemical analysis of the used materials. (wt%).

Material	SiO_2_	Al_2_O_3_	CaO	Fe_2_O_3_	MgO	Na_2_O	K_2_O	TiO_2_	*P*_2_O_5_	SrO	SO_3_	ZrO_2_	Cr_2_O_3_	MnO
*RM*	21.69	22.58	18.82	18.44	1.8	8.67	1.48	4.11	0.31	0.07	1.62	0.13	0.06	0.05
*P·O*	23.72	6.16	54.39	4.64	2.47	0.83	1.32	1.11	0.32	0.14	4.67	–	–	0.07
*BF*	52.02	12.58	8.06	6.42	6.05	5.89	5.69	2.33	0.77	0.2	–	–	–	–

### Mix proportion design

A preliminary experiment determined the water-to-binder ratio to be 0.35. In the main test, three volume replacement ratios (10%, 20%, and 30%)of cement (P·O) were selected to prepare supplementary cementitious materials, designated as R10, R20, and R30, respectively, while the control group without RM replacement was denoted as R0. The BF was incorporated into the cementitious matrix using an external mixing method. The content and length of BF were selected based on the characteristics and application conditions of the cement matrix, following the recommendations of the Chinese Standard GB/T 23,265 − 2009. Accordingly, four fiber lengths (6 mm, 9 mm, 12 mm, and 15 mm) and three fiber volume fractions (0.1%, 0.3%, and 0.5%) were employed. The volume fraction of BF is calculated based on the total volume of the cement paste (P·O + Water + RM). The mix proportions are summarized in Table 3.

**Table 3.** Mix proportion design.

Group number	*P*·O(kg/m^3^)	RM(kg/m^3^)	Water(kg/m^3^)	BF Length(mm)	BF Dosage(vol %)
R0	1300	0	455	0	0
R10	1170	130	455	0	0
R20	1040	260	455	0	0
R30	910	390	455	0	0
BFL6-BFC0.1	910	390	455	6	0.1
BFL6-BFC0.3	910	390	455	6	0.3
BFL6-BFC0.5	910	390	455	6	0.5
BFL9-BFC0.1	910	390	455	9	0.1
BFL9-BFC0.3	910	390	455	9	0.3
BFL9-BFC0.5	910	390	455	9	0.5
BFL12-BFC0.1	910	390	455	12	0.1
BFL12-BFC0.3	910	390	455	12	0.3
BFL12-BFC0.5	910	390	455	12	0.5
BFL15-BFC0.1	910	390	455	15	0.1
BFL15-BFC0.3	910	390	455	15	0.3
BFL15-BFC0.5	910	390	455	15	0.5

### Sample preparation

**Fig. 2:**
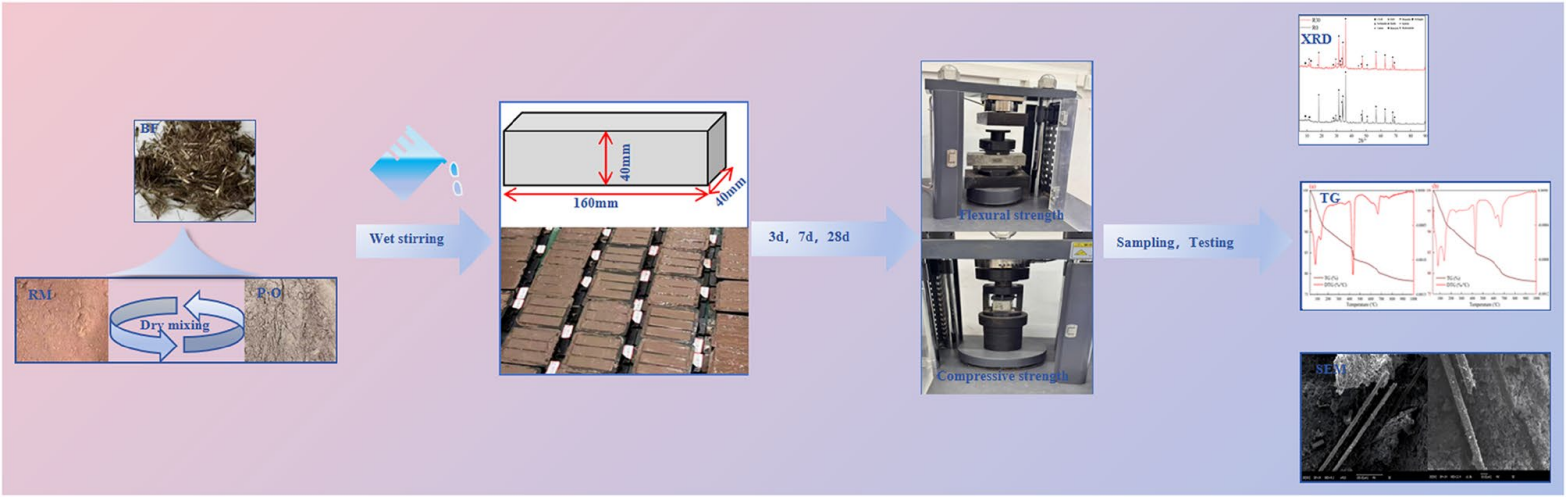
Sample preparation and test process.

The preparation and testing procedures of the samples are illustrated in Fig. 2. First, according to the mix proportions listed in Table 3, all raw materials were weighed by mass. To prevent agglomeration of BF within the cementitious matrix, cement, RM, and BF were first manually mixed for 1 min^52^. Then, water was added and mixed at a low speed for 120 s, followed by a 15 s pause during which the paste adhering to the mixing bowl and blades was scraped back into the center using a spatula. The mixture was then mixed again at a high speed for 120 s. After mixing, the slurry was poured into molds and compacted on a vibration table. The excess paste on the surface was leveled with a scraper. The specimens were demolded after 24 h of curing under ambient conditions and subsequently placed in a standard curing chamber until the specified curing ages, following the Chinese Standard GB/T 17,671 − 2021. Triple - linked mold of 40 × 40 × 160 mm were used for both compressive strength and flexural strength tests.

### Experimental methods

#### Mechanical property tests

The mechanical tests were conducted in accordance with the Chinese Standard GB/T 17,671 − 2021 at the Testing Center of the College of Civil Engineering, Guizhou University. A fully automatic microcomputer-controlled flexural and compressive testing machine (YAW-300 C) was used to determine the 3-day, 7-day, and 28-day flexural and compressive strengths (the applying rates was respectively 50 N/s and 2400 N/s). For each group, the flexural strength was taken as the average value of three specimens, while the compressive strength was calculated as the average of six measured values.

#### Characterization tests

The hydration products of R0 and R30 pastes were characterized by X-ray diffraction (XRD) and thermogravimetric analysis (TG). Samples were taken from the core regions of the 28-day compressive strength specimens. After drying in a vacuum oven for 24 h, the samples were ground into powder, sieved, and then subjected to XRD and TG tests. XRD measurements were performed using a Bruker D8 ADVANCE diffractometer (Germany) in conventional scanning mode at a rate of 2°/min. TG measurements were carried out using a METTLER TOLEDO TGA/DSC 3 + analyzer (Switzerland), with a temperature range from room temperature to 1000 °C and a heating rate of 10 °C/min. In addition, fragments (~ 5 mm) were taken from the central region of the freshly fractured surfaces of the failed specimens. The fragments were immersed in anhydrous ethanol for 1 day to terminate hydration, then dried in a 40 °C vacuum oven. The fracture surfaces were observed by scanning electron microscopy (SEM, EM-30, Korea) to investigate the failure mode at the fiber–matrix interface. 

For the quantitative XRD analysis, 15 wt% ZnO was introduced as an internal standard to improve the reliability of quantification and reduce the interference caused by Al2O3 from RM on diffraction intensities. Data processing was performed using HighScore Plus 4.9 software. Rietveld refinements followed the structural model fitting strategy proposed by Ray Young^53^, in which scale factors, zero shift, lattice parameters, and peak shape parameters were gradually adjusted to achieve optimal fitting with Rwp < 10. The standard reference cards employed were: ZnO (ICDD 01–083−6338), Portlandite (ICDD 98-020−2228), Calcite (ICDD 01–078−4614), Rutile (ICDD 04–016−0561), Ettringite (ICDD 04–022−3982), Hatrurite (ICDD 98-008−1100), Hematite (ICDD 98-008−2134), Katoite (ICDD 98-009−4631), Hydrotalcite (ICDD 04–011−5899), and Quartz (ICDD 01–075−8320). To evaluate the stability and reproducibility of the refinement results, each sample was independently refined three times, and the mean value of the three refinements was taken as the final quantitative result; the corresponding standard deviation and Rwp values were used to assess the statistical reliability of the analysis.

## Result and disscussion

### Effect of RM content on the mechanical properties

RM is a strongly alkaline industrial by-product generated during alumina refining^54^. Its fine particles and complex composition can interfere with the normal hydration and hardening processes of cement. Because RM possesses low intrinsic cementitious activity, its direct incorporation into cement-based materials without modification generally exerts an adverse effect on mechanical performance, often leading to reduced strength^55^.

**Fig. 3:**
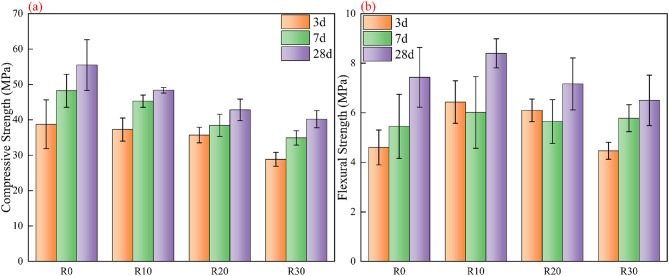
Evolution of mechanical performance of red-mud-blended cement pastes at different curing ages. (**a**) Compressive strength; (**b**) Flexural strength at 3, 7, and 28 days.

As shown in Fig. 3a, the compressive strength decreases overall with increasing RM content. At the 3-day curing age, incorporating 10% RM had little influence on compressive strength, and the specimens showed values comparable to those of the RM-free control group. When the RM content reached 30%, a noticeable decline in early-age compressive strength was observed. With extended curing to 7 days and 28 days, the negative influence of RM became more pronounced: the 30% RM group exhibited only about 70% of the control group’s 28-day compressive strength, dropping from 55.5 MPa to 40.2 MPa, representing roughly a 30% reduction.

By contrast, the 10% and 20% RM mixtures showed slightly lower 28-day strengths than the control but within the range of experimental deviation, indicating that low to moderate RM dosages caused only a mild reduction in compressive strength. Overall, as the RM content increased, the effective cementitious components were diluted and the low reactivity of RM restricted the formation of hydration products, leading to a continuous strength loss. This tendency agrees with previous studies, which attribute strength reduction to the dilution effect and poor pozzolanic activity of RM^56,57^.

The variation of flexural strength with RM content exhibited a different trend from that of compressive strength, as shown in Fig. 3b. At the early curing stage, specimens containing a small amount of RM even showed slightly higher flexural strength than the control group. This could be attributed to the fine RM particles filling the microvoids within the cement matrix and providing nucleation sites^15^, which enhanced the early-stage flexural performance^58^.

Even at 7 days, the flexural strengths of all RM-containing specimens remained comparable to that of the control group, showing no noticeable reduction. After 28 days of curing, the flexural strength of each group further increased: the 10% RM mixture achieved the highest value, slightly above the control, the 20% RM group showed a similar level, while the 30% RM group exhibited a slight decrease. These results indicate that flexural strength is less sensitive to RM content. A small addition of RM does not significantly weaken, and may even slightly enhance, the flexural performance at certain curing ages. Only when the RM content reached 30% did a minor decline in flexural strength appear.

In summary, increasing the RM content generally reduced the mechanical performance of the cement-based materials, with the reduction in compressive strength being particularly evident. Therefore, subsequent studies on fiber reinforcement were carried out based on specimens containing 30% RM, aiming to improve their mechanical properties through modification.

### Effect of fibers on the flexural strength

The age-specific two-way ANOVA summary for flexural strength, including the overall F and P values for BFL, BFC, and BFL × BFC, is provided in Table 4. Detailed ANOVA outputs and multiple-comparison results for each curing age are provided in the Supplementary Material. At the 3-day curing age, neither fiber length nor fiber content exhibited a significant main effect on the flexural strength of the specimens, and the interaction between the two factors was also insignificant. By 7 days, the main effect of fiber length became significant, and a notable interaction between fiber length and fiber content appeared. This indicates that the influence of fiber length on flexural strength at this stage depended on the level of fiber content. However, the main effect of fiber content at 7 days remained statistically insignificant. With a further extension of curing to 28 days, the main effect of fiber content became significant, while the effect of fiber length was no longer evident, and their interaction also showed no statistical significance. These findings suggest a time-dependent evolution in the influence of fibers on the flexural performance of RM–cement specimens: initially negligible at early age, dominated by fiber length and its interaction with content at the middle stage, and primarily governed by fiber content at later ages.

**Fig. 4:**
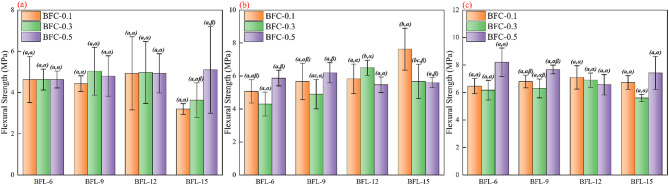
Variations in flexural strength of samples with different BFL and BFC at curing ages of 3, 7, and 28 days. (**a**) 3 days; (**b**) 7 days; (**c**) 28 days. a, b, c indicates significant differences among BFLs at the same BFC level (p ≤ 0.05), while α, β indicate significant differences among BFCs at the same BFL level (p ≤ 0.05).

Figure 4 presents the corresponding flexural strength values with error bars together with the grouping letters from the post-hoc pairwise comparisons. As shown, different combinations of fiber length and fiber content exhibited distinct stage-dependent effects on the development of flexural strength. At the early curing stage, the strength values of all specimens were generally similar, and the influence of fibers was not yet evident. Neither increasing fiber length nor fiber content produced a noticeable improvement in 3-day strength, as the differences among groups were mostly within the experimental error range, maintaining an overall level of approximately 4.63 MPa. By 7 days, the reinforcing effect of fibers became apparent. At a fiber content of 0.1%, increasing fiber length slightly enhanced the strength, with the 15 mm group showing a relatively higher value of 7.63 MPa. When the fiber content was 0.3%, the 12 mm fibers yielded the highest strength (6.50 MPa), while both shorter and longer fibers resulted in slightly lower values, indicating the existence of an optimal fiber length. At a higher fiber content of 0.5%, the trend changed: the 9 mm fiber group reached a strength of 6.20 MPa, whereas excessive fiber length led to a reduction in strength to around 5.50 MPa. At the 28-day curing age, the differences in strength among fiber combinations became more pronounced. For the 0.1% fiber content, the trend of moderate fiber length being more favorable continued, while overly long fibers caused a slight decrease in strength. At 0.3% fiber content, fiber length had a more distinct effect: the 12 mm group achieved the highest strength of 6.90 MPa, whereas the 15 mm group showed a clear reduction to 5.60 MPa. In contrast, under the 0.5% fiber content condition, shorter fibers performed better, the 6 mm fiber group reached the highest 28-day strength of 8.20 MPa, while the 12 mm fibers gave the lowest strength among all combinations.

According to the results of the two-way ANOVA and Table S1-S3 discussed above, the effects of fiber length and fiber content on the flexural strength of RM–cement specimens exhibited distinct stage-dependent characteristics at different curing ages. At the 3-day age, the contribution of both factors to flexural strength was limited. The main effects of fiber length and content were not statistically significant (*P* > 0.05), and no significant interaction between them was observed. This indicates that, at the early curing stage, the addition of fibers did not yet produce a meaningful improvement in flexural capacity. The strength values among different combinations fluctuated only slightly and showed minimal enhancement compared with the fiber-free control. This can be attributed to the insufficient degree of hydration in the cement matrix, which results in an underdeveloped interfacial bond between the fibers and the matrix. Consequently, the fibers were unable to effectively bear tensile stresses at this stage, leading to a lack of statistically significant reinforcement. By 7 days, the statistical analysis revealed that the effects of both fiber length and content became significant, as reflected by higher F-values and P-values below 0.05. Notably, a significant interaction between the two factors emerged, suggesting that the improvement in flexural strength varied markedly among different combinations of fiber parameters, indicating a synergistic effect. As illustrated in Fig. 4, the flexural strength increased distinctly with both fiber length and content, though this increase was not linearly additive. Instead, it depended on the proper coordination between the two parameters: fibers of suitable length at an appropriate dosage led to a substantial enhancement in 7-day strength compared with the reference group, whereas excessively short fibers at high dosages or overly long fibers at low dosages produced little improvement. This is further supported by the dispersion observed among the 7-day results, specimens with the optimal fiber combination showed a marked strength gain over the fiber-free sample, while suboptimal combinations exhibited much smaller improvements, confirming the presence of a significant interaction effect. At the 28-day age, the reinforcing effect of fibers persisted but displayed a different statistical pattern. Both fiber length and content remained significant main factors, indicating that each continued to contribute independently to the later-age strength. However, the interaction term approached the threshold of significance (*P* ≈ 0.05), reaching only a marginally significant level. Compared with the 7-day results, the differences among various fiber combinations at 28 days became smaller. Although the optimal combination still achieved the highest flexural strength, its relative advantage over other groups diminished. This suggests that as curing progressed and the cement matrix became denser and stronger overall, the additional strength gain resulting from optimized coordination between fiber length and content decreased. In other words, the synergistic interaction between fiber parameters was less pronounced at later ages than during the intermediate curing stage.

**Table 4.** Summary of the age-specific two-way ANOVA results for flexural strength: overall effects of BFL, BFC, and BFL × BFC at 3, 7, and 28 days (F and P values).

	3D(F, *P*)	7D(F, *P*)	28D(F, *P*)
BFL	(1.221,0.324)	(3.822,0.023)	(0.482,0.698)
BFC	(0.742,0.487)	(2.411,0.111)	(9.054,0.001)
BFL×BFC	(0.586,0.738)	(3.356,0.015)	(2.205,0.078)

### Effect of fibers on the compressive strength

The age-specific two-way ANOVA summary for compressive strength, including the overall F and P values for BFL, BFC, and BFL × BFC, is provided in Table 5. Detailed ANOVA outputs and multiple-comparison results for each curing age are provided in the Supplementary Material. Unlike flexural strength, both fiber length and fiber content exhibited statistically significant main effects on the compressive strength of the specimens at the 3-day curing age, while their interaction was not significant. With the curing age extended to 7 days, the main effects of fiber length and fiber content remained significant, and their interaction also reached a statistically significant level at this stage. By 28 days, the main effect of fiber length was still significant, whereas the effect of fiber content was no longer significant, and their interaction was again found to be insignificant. These results indicate that as curing progressed, the influence of fiber length on compressive strength remained consistently significant, while the influence of fiber content gradually weakened and became non-significant at 28 days. Moreover, the interaction between fiber length and fiber content was significant only at the intermediate curing stage.

**Fig. 5:**
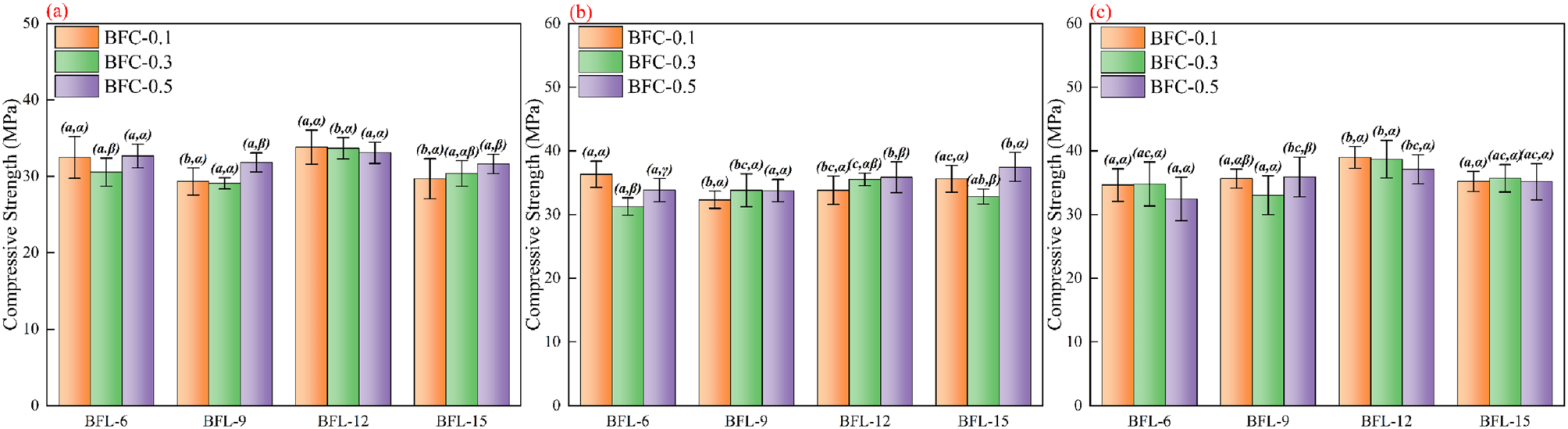
Variations in compressive strength of samples with different BFL and BFC at curing ages of 3, 7, and 28 days. (**a**) 3 days; (**b**) 7 days; (**c**) 28 days. a, b, c indicates significant differences among BFLs at the same BFC level (p ≤ 0.05), while α, β, γ indicate significant differences among BFCs at the same BFL level (p ≤ 0.05).

Figure 5 presents the corresponding compressive strength values with error bars together with the grouping letters from the post-hoc pairwise comparisons. The results show that the compressive strength of all fiber-reinforced cement specimens increased markedly with age. In contrast to the behavior observed for flexural strength, the differences among the combinations of fiber length and content displayed a distinct stage-dependent pattern. At the 3-day age, the compressive strength was relatively low when the fiber content was 0.3%, while a slight increase was observed when the content was raised to 0.5%, indicating a certain compensating effect. For specimens with a fiber length of 12 mm, the compressive strengths across different contents were the highest and exhibited minimal variation, with average values remaining above 33 MPa. By 7 days, the results became more complex. For the 6 mm fibers, the strength first decreased from 36.32 MPa at 0.1% content to 31.25 MPa at 0.3%, then rose again to 33.82 MPa at 0.5%, showing considerable fluctuation. At 9 mm and 12 mm fiber lengths, compressive strength generally followed a monotonically increasing trend with higher fiber content. However, at 15 mm, the 0.5% group reached the highest strength of 37.45 MPa, while the 0.3% group was noticeably lower, reflecting the complex interaction between fiber length and content. At the 28-day curing age, the main effect of fiber length remained significant, whereas those of fiber content and their interaction were no longer significant. The compressive strengths of all groups continued to increase, and the differences among them became smaller. For the 12 mm fiber length, all content groups maintained a relatively high and stable strength of around 38 MPa, demonstrating excellent compatibility between fiber and matrix. For the 15 mm fibers, although intergroup differences were also small, the overall compressive strength decreased to approximately 35 MPa. When the fiber length was shorter than 12 mm, increasing the fiber content resulted in a reduction in compressive strength, indicating that excessive fiber dosage could adversely affect the matrix integrity at shorter lengths.

Based on the ANOVA results and Table S4 -S6, it can be seen that the effects of fiber length, fiber content, and their interaction on compressive strength exhibited a pronounced age dependence and a stage-wise evolution. At the 3-day curing age, both fiber length and fiber content showed significant main effects, whereas their interaction was not significant (*P* = 0.131). Further pairwise comparisons revealed that compressive strength generally reached a local minimum when the fiber content was 0.3%, though it is noteworthy that fibers with a length of 12 mm maintained a stable strength of around 33 MPa across all content levels. This indicates that at the early stage of setting and hardening, fiber length played a more crucial role in bridging initial microcracks and transferring stress, while the influence of fiber content was primarily driven by specific length levels without systematic coupling between the two parameters—hence, the interaction was not statistically significant. By 7 days, both fiber length and fiber content became significant main effects, and their interaction reached a highly significant level. As shown in Fig. 5, short fibers exhibited a decrease–increase trend in compressive strength with increasing content, while long fibers showed an overall increasing but non-monotonic pattern. This confirms the existence of a strong interaction effect. At this stage, the hydration process and interfacial bonding were sufficient to support the formation of an effective fiber network^59^. However, short fibers at medium content tended to suffer from dispersion deterioration and defect accumulation, whereas longer fibers required higher dosages to establish a continuous bridging network, leading to pronounced performance differences among combinations. At the 28-day curing age, the main effect of fiber length remained significant, while those of content and interaction became insignificant. The 12 mm fibers consistently yielded the highest compressive strengths with minimal variation across different contents. This suggests that as curing progressed, the densification of the matrix and the maturation of the interfacial transition zone weakened the marginal contribution of fiber content to load-bearing performance. Consequently, its explanatory power in variance analysis was substantially diluted. In contrast, the factor of “effective fiber length”- which determines whether fibers can effectively bridge characteristic crack intervals and delay crack coalescence before compressive failure- remained dominant. In summary, the evolution of the statistical characteristics followed a clear sequence: at the early stage, fiber length dominated while content played a secondary and independent role; at the middle stage, both factors acted jointly with strong interaction; and at the late stage, fiber length became the sole dominant factor as the effects of content and interaction diminished. This progression was fully consistent with the pattern of mean differences indicated by the significance labels in both rows and columns of the statistical tables.

Overall, when combined with the findings in Sect. 3.2, the combination of 12 mm fiber length and 0.1% fiber content was identified as the best overall-performing mixture among the investigated fiber-reinforced 30% RM specimens. Although its 28-day compressive strength of 38.95 MPa was slightly lower than that of the fiber-free R30 specimen of 40.20 MPa, it improved the flexural strength by 0.6 MPa and maintained a relatively high and stable compressive response. At this configuration, the fiber length closely matched the critical fiber length and characteristic crack spacing of the system, providing sufficient bridging and anchorage to enhance load transfer without significantly impairing mixing uniformity or matrix continuity. In contrast, excessive fiber length or content intensified fiber–fiber interactions, leading to entanglement, poor orientation, and agglomeration. These effects increased porosity and microdefects, weakened the interfacial transition zone, and offset the bridging benefits, resulting in a decline in overall strength.

**Table 5.** Summary of the age-specific two-way ANOVA results for compressive strength: overall effects of BFL, BFC, and BFL × BFC at 3, 7, and 28 days (F and P values).

	3D(F, *P*)	7D(F, *P*)	28D(F, *P*)
BFL	(13.364, 0)	(4.657,0.005)	(8.901,0)
BFC	(3.791,0.028)	(5.837,0.005)	(0.797,0.455)
BFL×BFC	(1.722,0.131)	(5.627,0)	(1.271,0.284)

### The influence of red mud on cement paste hydration

#### QXRD analysis

**Fig. 6:**
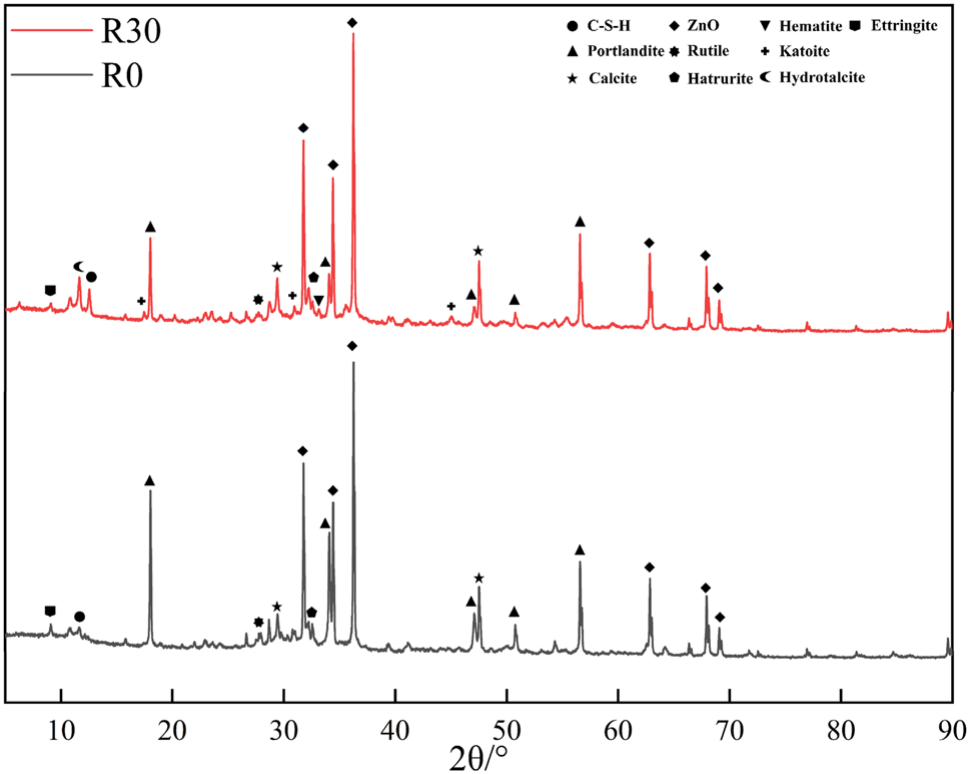
XRD pattern of R0 and R30.

The mineral phase compositions of the samples are listed in Table 6 and the XRD pattern are shown in Fig. 6. After incorporating 30% RM, the amorphous phase increased from 6.16% to 19.98%, while the proportion of crystalline phases decreased from 93.84% to 80.02%, indicating that RM reduced the overall crystallinity of the system. This also suggests that RM altered the hydration pathway, favoring the formation of low-order C–S–H or C–A–S–H gels and certain poorly diffracting Al- and Mg-rich hydrated phases, thereby increasing the proportion of the unresolvable matrix. The variations in crystalline hydration phases further corroborate this conclusion. The content of portlandite (Ca(OH)_2_) decreased from 11.17% to 5.92%, reflecting a reduction in available calcium sources due both to the dilution of active clinker and to the consumption of Ca by reactive aluminosilicate species that became incorporated into gels or secondary hydrates. Meanwhile, ettringite (AFt) dropped sharply from 30.22% to 1.20%, suggesting that the high-alkalinity, high-alumina environment induced by RM strongly inhibited the crystallization of sulfate–aluminate phases. The content of tricalcium silicate (C_3_S) decreased from 37.44% to 30.65%, which corresponds to the reduction of clinker minerals caused by partial cement replacement and indicates that RM addition did not significantly affect the hydration of C_3_S. In contrast to the above reductions, the RM-blended cement paste contained 21.37% hydrotalcite and 3.60% katoite. The formation of these phases demonstrates that Mg and Al supplied by RM participated in the hydration reactions under strong alkaline conditions, inhibiting the crystallization of AFt and promoting the development of layered or garnet-type structures through their combination with carbonate and silicate anions. This process also transformed part of the Al and Ca phases into low-order gel networks. The presence of minor hematite residues in the RM-modified sample further supports this interpretation. Overall, these hydration changes explain the limited strength development and reduced stiffness observed in the RM system at 28 days. Regarding carbonation, the calcite content increased from 1.60% to 3.68%, accompanied by substantial formation of hydrotalcite. Since hydrotalcite commonly incorporates CO_3_^2−^ as interlayer anions, this indicates that carbonate species were effectively immobilized within the layered structure. It should be emphasized, however, that high alkalinity is not the direct driving force of carbonation; carbonation is governed by the supply and diffusion of external CO_2_. The highly alkaline pore solution merely facilitates chemical absorption of CO_2_ at the material surface and enhances carbonate fixation.

From the XRD results, the RM-containing system at 28 days mainly exhibited local carbonate incorporation and interlayer entrapment within layered phases, without signs of abnormal calcite enrichment or intensive carbonation. In summary, the incorporation of RM reshaped the phase equilibrium and crystallinity of hydration products by increasing the amorphous matrix fraction, suppressing the crystallization of key hydrated phases, and promoting the dominance of hydrotalcite and katoite. This microstructural transformation is the fundamental mechanism underlying the deterioration in mechanical performance of RM-modified cement pastes.

**Table 6.** XRD Rietveld refinement results of 28-day cement pastes with and without 30% red mud. (wt%).

	R0	R30
Amorphous hydrate	6.15 ± 0.13	19.98 ± 1.21
ZnO	13.04	13.04
Ca(OH)_2_	11.17 ± 0.14	5.92 ± 0.75
CaCO_3_	1.60 ± 0.26	3.68 ± 0.67
Rutile	0.38 ± 0.02	–
Ettringite	30.22 ± 1.18	1.20 ± 0.08
Hatrurite	37.44 ± 1.19	30.65 ± 1.63
Hematite	–	0.56 ± 0.01
Katoite	–	3.60 ± 0.08
Hydrotalcite	–	21.37 ± 0.33

#### Thermal analysis

**Fig. 7:**
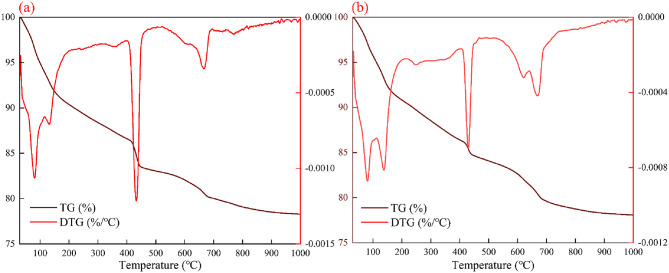
TG and DTG curves of samples. (**a**) R0; (**b**) R30.

The results presented in Fig. 7; Table 7 show that the incorporation of RM redistributes low-temperature bound water, weakens the dehydroxylation of portlandite at medium temperature, and enhances the two-step decarbonation at high temperature. The low-temperature region consists of STAGE 1 and STAGE 2. For the plain cement sample, the weight losses were 4.53% and 2.90%, respectively, giving a total of 7.43%, which corresponds to the release of pore water and weakly bound water, along with the removal of low-temperature structural water from AFt and C–S–H phases. After the addition of 30% RM, the weight loss in STAGE 1 slightly decreased to 4.12%, whereas that in STAGE 2 increased markedly to 4.58%. The DTG peak shifted upward to 138.01 °C and extended toward higher temperatures, with a combined weight loss of 8.71%. This low-temperature behavior indicates that RM reduced the fraction of crystalline bound water while promoting the retention of water molecules within interlayer and framework sites of C–A–S–H and Mg–Al layered phases, leading to an upward shift of DTG peaks and a higher total weight loss. The medium-temperature region corresponds to the dehydroxylation of Ca(OH)_2_. In the plain cement paste, the weight loss in this region was 3.35%; after RM incorporation, the temperature range narrowed and the loss decreased to 1.70%. Based on the water loss fraction from CH dehydroxylation, the estimated mass fraction of CH declined from approximately 13.8% to 7.0%, consistent with the XRD results showing a reduction in Ca(OH)_2_ from 11.17% to 5.92%. This confirms that RM addition consumed part of the calcium source and reduced the buffering capacity of the pore solution alkalinity. The high-temperature region was dominated by the two-step decarbonation processes (STAGE 4 and STAGE 5). For the plain cement sample, the corresponding thermal losses were 0.73% and 1.55%, whereas for the RM-blended sample, they increased to 1.79% and 1.83%, respectively. The STAGE 4 peak also shifted to higher temperatures, suggesting that the carbonates were embedded in a more stable microenvironment, stabilized by cation substitution or encapsulation within the matrix. According to the weight loss ratio from CaCO_3_ decomposition, the equivalent carbonate content increased from approximately 5.2% to 8.2%, slightly exceeding the fraction of crystalline calcite detected by XRD. This implies that a considerable portion of carbonate species was dispersed and fixed within hydrotalcite, layered aluminates, or gel frameworks.

Overall, the cumulative weight loss from STAGE 1 to STAGE 5 increased from 13.05% to 14.03%, characterized by an increase in low-temperature losses, attenuation of CH-related features at medium temperature, and enhancement of high-temperature decarbonation. The thermal evolution established by TG–DTG analysis aligns well with the phase composition evidence from XRD: RM drives the redistribution of water and anions from crystalline hydrates dominated by AFt and CH to disordered and layered storage modes centered on C–A–S–H and layered double hydroxides. Simultaneously, RM enhances the dual fixation and thermal stability of carbonate species. These transformations represent the thermal origin of the lower crystallinity and weakened alkalinity buffering capacity observed in the RM-containing system at 28 days.

**Table 7.** Thermal decomposition stages, mass loss percentages, and corresponding DTG peak temperatures of samples.

		STAGE 1	STAGE 2	STAGE 3	STAGE 4	STAGE 5
R0	Weight loss (wt%)	4.53	2.90	3.35	0.73	1.55
	Peak (℃)	80.52	131.77	433.96	603.34	667.87
R30	Weight loss (wt%)	4.12	4.58	1.70	1.79	1.83
	Peak (℃)	81.15	138.01	429.62	622.02	668.69

### Macro-and micro-fracture characteristics and reinforcement mechanism analysis

**Fig. 8:**
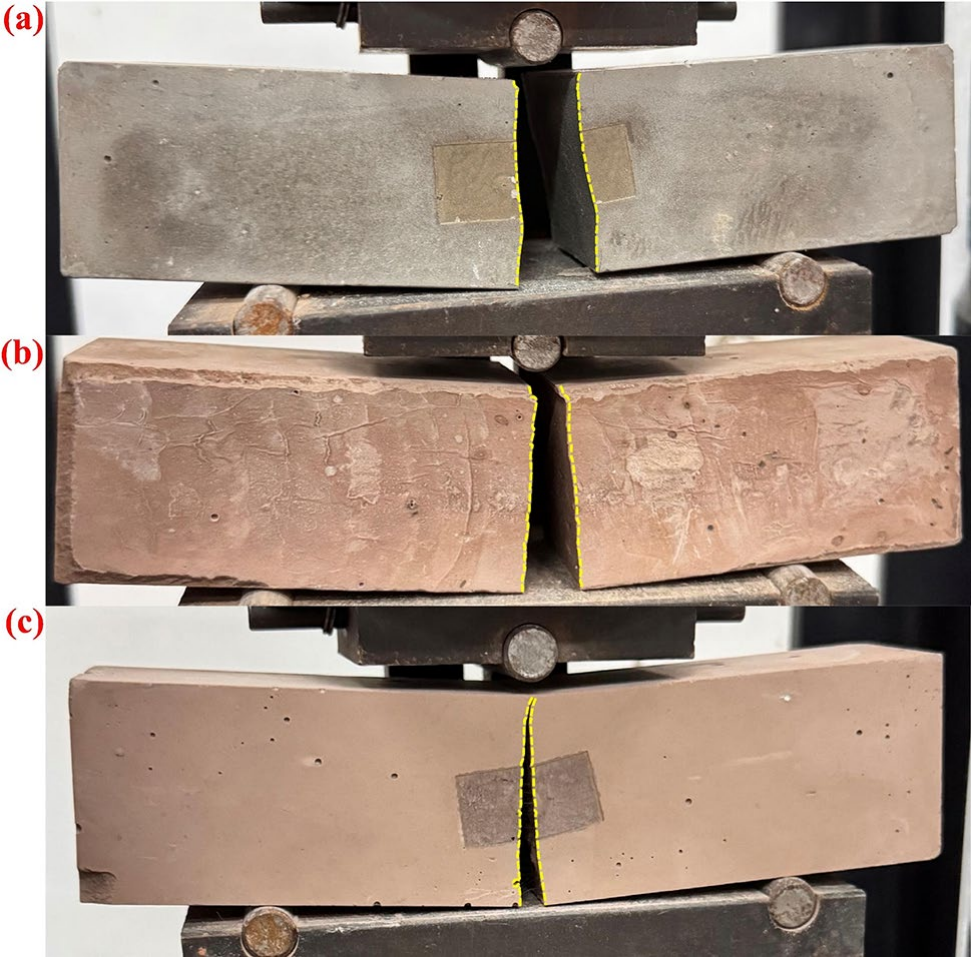
Failure modes of 28 d flexural specimens. (**a**) R0;(**b**) R30; (**c**) BFL12-BFC0.1.

Since the cement paste matrix contains no coarse or fine aggregates, it lacks the typical crack-arresting network formed by the combined action of aggregates and fibers in conventional concrete. Therefore, the toughening mechanism in this system mainly relies on the intrinsic bridging effect of the fibers themselves^46^. For BF reinforced cement paste specimens containing 30% RM, the macroscopic fracture morphology showed a high degree of consistency. As illustrated in Fig. 8, the fractures were generally characterized by a single major crack running through the central region of the specimen, with relatively flat and smooth fracture surfaces. The cracks were slightly inclined and rarely exhibited secondary branching. This indicates that, unlike in concrete, the absence of aggregate-fiber synergy, combined with the use of untreated short-cut BF, resulted in easy fiber pull-out during flexural cracking, thereby limiting the crack-bridging and crack-arresting effects. Consequently, the improvement in flexural strength was minimal, consistent with both the observed fracture morphology and the mechanical test results.

**Fig. 9:**
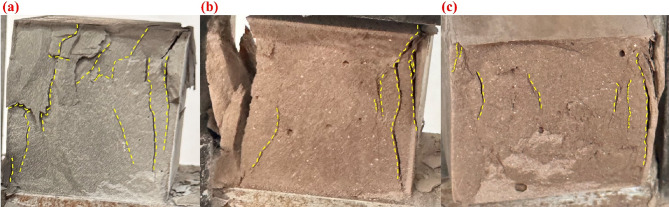
Failure modes of 28 d compressive specimens. (**a**) R0; (**b**) R30; (**c**) BFL12-BFC0.1.

Under compressive loading, BF effectively altered the failure pattern of the cement paste specimens. As shown in Fig. 9, the fiber-free samples developed large penetrating cracks and fragmented catastrophically after reaching the peak load. In contrast, the specimen with 12 mm fibers at a dosage of 0.1% exhibited fewer visible cracks and better retained its overall integrity after failure. This suggests that the principal role of fiber incorporation in the present system was to improve crack restraint and post-failure stability, rather than to maximize the peak compressive strength alone. Conversely, when the fiber length was too short or the fiber content too low, the fibers were insufficient to effectively restrain crack propagation, leading to the formation of through-cracks and rapid disintegration. With further increases in fiber length or content, the specimens became more confined internally by the fiber network. Large cracks were replaced by finer ones, the fragment size decreased, and the crack distribution became denser and more complex. The bridging and stress-dispersion functions of the fibers promoted the initiation of multiple microcracks and limited their propagation, preventing abrupt failure caused by a single dominant crack. However, excessive fiber dosage^60,61^ could lead to poor dispersion and defect formation, thereby weakening the internal bonding of the cement matrix and reducing mechanical strength. Overall, BF improved the ductility and energy absorption capacity^43,62^ of the cement paste under compression by suppressing macroscopic through-crack development and inducing the formation of a fine, distributed microcrack network.

**Fig. 10:**
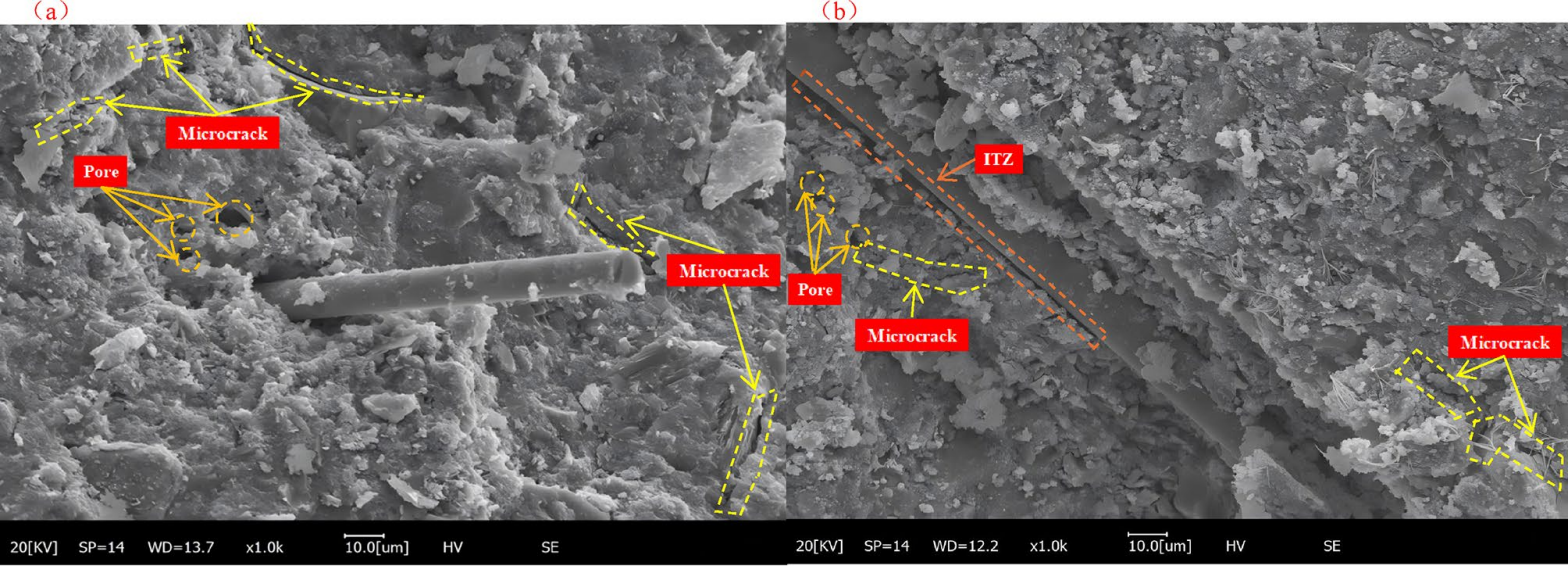
SEM images of Paste and Fibers -Matrix ITZ. (**a**) Paste; (**b**) Fibers -Matrix ITZ.

Microscale fracture analysis further elucidated the fiber reinforcement mechanism. As shown in Fig. 10, microcracks exist in the red mud–cement slurry system, and obvious pores are present on the matrix surface. Due to the physical filling effect of RM particles, the overall structure is dense^63^, These defects are the main factors affecting the slurry performance. The interfacial transition zone (ITZ) is the weak link between fibers and the matrix, and its microstructural characteristics directly influence the macroscopic mechanical properties^64^. Moreover, the incorporation of fibers leads to the formation of ITZ between fibers and the matrix, increasing the porosity and water absorption of the slurry.

**Fig. 11:**
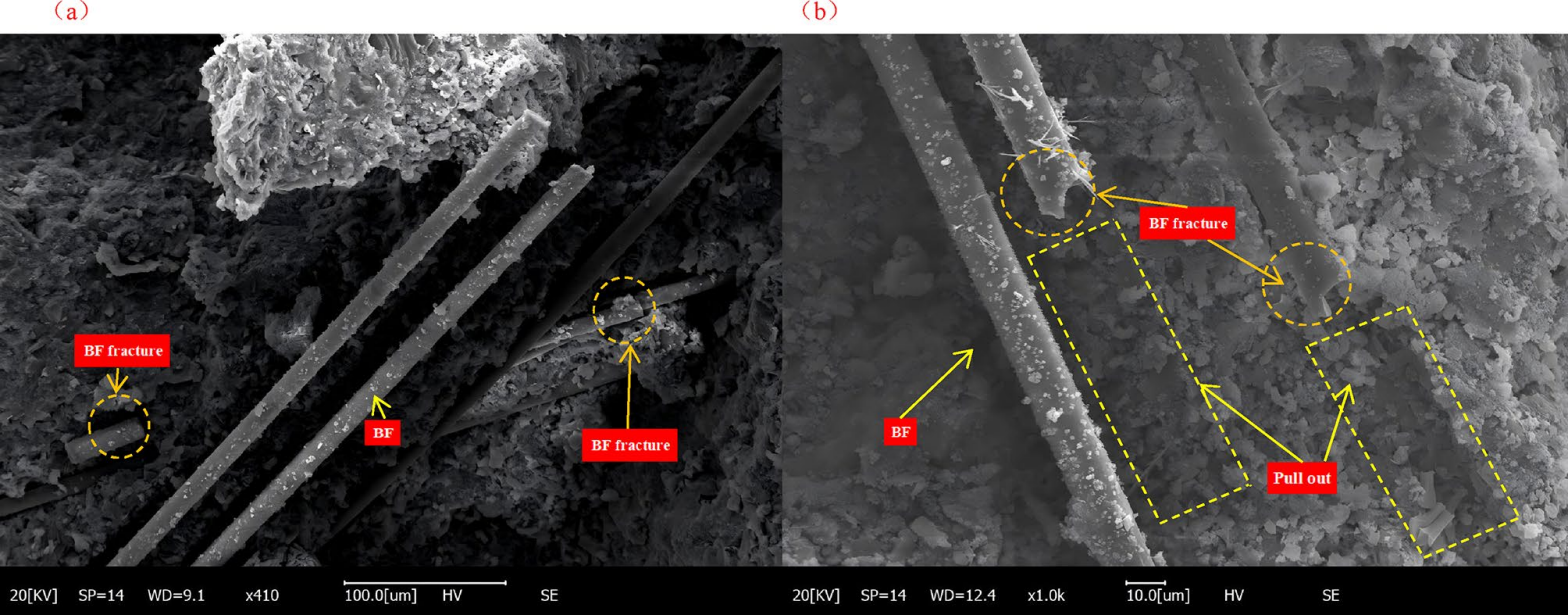
SEM images showing the morphology and distribution of basalt fibers in specimens. (**a**) Fibers embedded inside the matrix; (**b**) Fibers observed on the fractured surface after splitting.

**Fig. 12:**
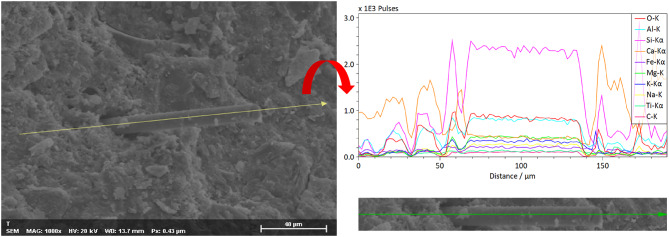
SEM-EDS line-scan elemental distribution map of the interface between basalt fiber and red mud-cement matrix.

As shown in Fig. 11, numerous BF were observed to have been pulled out from the fracture surface. The pull-out lengths varied among fibers, and most fiber ends remained intact, indicating that interfacial debonding and slippage occurred between the fibers and the matrix during crack propagation, thereby dissipating fracture energy. Meanwhile, a few fibers were found to have fractured, suggesting that local stresses in certain regions reached the intrinsic tensile strength of the fibers. This phenomenon helps explain the partial improvement in flexural strength observed in some specimens. Notably, prismatic crystalline hydration products were attached to the surfaces of some pulled-out fibers, and remnants of cement paste were adhered to them. This demonstrates the presence of interfacial bonding between the fibers and the cement matrix—the fiber surfaces were covered by hydration products, reflecting a good early-stage interfacial connection. Furthermore, since BF are rich in SiO₂, surface dissolution reactions may occur under the highly alkaline conditions of the cement paste, leading to the formation and deposition of C–S–H gel on the fiber surface. It can therefore be inferred that BF might have participated partially in the cement hydration process, forming an interlocking bond at the fiber- matrix interface. This interfacial interlocking improved adhesion and stress-transfer efficiency at the microscale. Under the combined influence of macroscopic and microscopic mechanisms, the physical bridging and interfacial bonding of the fibers worked synergistically to enhance both the strength and toughness of the composite. This synergy effectively impeded the rapid coalescence of cracks, thereby improving the overall mechanical performance and fracture resistance of the cement paste containing 30% RM. As shown in Fig. 12, SEM-EDS line-scan analysis was performed on a 12 mm fiber-reinforced red mud-cement paste. The scanning path was parallel to the axial surface of the fiber, starting from the interior of the fiber, passing through the fiber–matrix interface, and extending into the distant matrix. In the fiber region, the Si-element signal intensity was highest and remained stable, which is entirely consistent with the chemical composition of basalt fiber where SiO₂ is the main component. Upon entering the matrix region, the Si signal intensity dropped significantly but still remained at a relatively high level, corresponding to the cement hydration products (C-S-H gel) and partly unhydrated tricalcium silicate (C₃S) and dicalcium silicate (C₂S) particles. The distribution trend of Ca was almost a mirror image of that of Si: in the fiber region, the Ca-element signal intensity was very low, reflecting its low content in the fiber. In the matrix region, however, the Ca intensity was very high, indicating the presence of calcium-rich C-S-H gel and abundant calcium hydroxide. The SEM-EDS results reveal that chemical reactions occurred in the ITZ region. The reactive silica on the surface of the basalt fiber participated in the hydration reaction to form C-S-H gel, thereby enhancing the interfacial bond strength.

## Conclusion

This study systematically investigated the high-content utilization of red mud in cement paste and its synergistic reinforcement through fibers. Red mud replacement ratios of 10%, 20%, and 30% were set, with 30% used as the matrix for a full-factorial design of basalt fiber parameters. Fiber lengths of 6, 9, 12, and 15 mm were combined with volume fractions of 0.1%, 0.3%, and 0.5%. Compressive and flexural strengths were tested at 3, 7, and 28 days, and microstructural characterizations were integrated to establish correlations among mix parameters, mechanical performance, and structural evolution. The conclusions reached are as follows:

1. Incorporating 30% red mud reduced the 28-day compressive strength from 55.50 MPa to 40.20 MPa, while its effect on flexural strength was relatively minor. Mechanistically, the addition of red mud altered the hydration pathway, shifting from AFt-dominated crystalline hydrates toward amorphous phases and layered hydrotalcite. The reductions in portlandite and ettringite, accompanied by intensified carbonation, promoted the formation of hydrogarnet and calcite. Consequently, the crack nucleation threshold decreased, leading to concurrent degradation of load-bearing capacity and toughness. Basalt fibers form a reactive interfacial transition zone where fiber-surface silica partially dissolves to form C-S-H gel, enhancing bonding and enabling effective crack bridging, though the increased porosity around fibers also introduces new interfacial defects.
2. With increasing curing age, the fiber effect evolved in stages. At 3 days, fiber length and dosage jointly governed compressive strength but had limited influence on flexural behavior. At 7 days, their synergistic interaction produced a combined dependency. By 28 days, compressive strength was primarily controlled by fiber length, whereas flexural strength depended mainly on dosage, reflecting the maturation of the interfacial transition zone and the stabilization of characteristic crack scales. Among the investigated fiber-reinforced mixtures, the combination of 12 mm fiber length and 0.1% dosage provided the best overall balance of mechanical performance. Although this mixture did not yield the highest absolute compressive strength relative to the fiber-free R30 specimen, it improved the flexural strength by 0.6 MPa, maintained a relatively high and stable compressive response, and exhibited superior failure integrity.
3. Fiber reinforcement relied on the synergistic effects of crack-bridging, pull-out energy dissipation, and interfacial interlocking. Bridging suppressed main-crack propagation and promoted crack refinement; fiber pull-out and occasional rupture provided sustained energy absorption to delay post-peak instability; and interfacial bonding ensured efficient load transfer. The incorporation of red mud lowered the matrix crystallinity and increased layered phases, while the combination of 12 mm length and 0.1% dosage most effectively formed a well-dispersed bridging network spanning characteristic cracks. This configuration achieved dual compensation for strength and toughness without altering the chemical environment of the matrix. Deviations from this window led to poor fiber orientation, agglomeration, and interfacial weakening, offsetting the reinforcement benefits.

These insights provide an operable mix-design window and microstructural foundation for the resource utilization of red mud and optimization of fiber parameters.

## Supplementary Information

Below is the link to the electronic supplementary material.

Supplementary Material 1

## Data Availability

The datasets used and/or analyzed during the current study are available from the corresponding author on reasonable request.
