# Supplementary material for: Mechanism of the influence of basalt fiber parameters on the mechanical properties and microstructure of high-volume red mud-cement paste
Source: Sci Rep. 2026 Apr 10;16:16602. doi: 10.1038/s41598-026-47848-1 (PMC13219395; doi:10.1038/s41598-026-47848-1)
Supplement: Supplementary file 1 — Supplementary Material 1 [file 41598_2026_47848_MOESM1_ESM.docx]

Table S1. Full two-way ANOVA output for flexural strength at 3 days.

|  | BFC-0.1 | BFC-0.3 | BFC-0.5 | F | P |
| --- | --- | --- | --- | --- | --- |
| BFL-6 | 4.63±1.12(a,α) | 4.63±0.51(a,α) | 4.63±0.41(a,α) | 0 | 1 |
| BFL-9 | 4.43±0.38(a,α) | 5.03±1.16(a,α) | 4.80±0.98(a,α) | 0.211 | 0.811 |
| BFL-12 | 4.93±1.77(a,α) | 4.97±1.50(a,α) | 4.93±0.95(a,α) | 0.001 | 0.999 |
| BFL-15 | 3.2±0.26(a,α) | 3.63±0.84(a,αβ) | 5.10±2.10(a,β) | 2.288 | 0.123 |
| F | 1.338 | 0.964 | 0.091 |  |  |
| P | 0.285 | 0.426 | 0.965 |  |  |

Table S2. Full two-way ANOVA output for flexural strength at 7 days.

|  | BFC-0.1 | BFC-0.3 | BFC-0.5 | F | P |
| --- | --- | --- | --- | --- | --- |
| BFL-6 | 5.07±0.71(a,αβ) | 4.3±0.72(a,α) | 5.87±0.47(a,β) | 2.891 | 0.075 |
| BFL-9 | 5.67±1.10(a,αβ) | 4.90±0.89(ac,α) | 6.20±0.61(a,β) | 2.011 | 0.156 |
| BFL-12 | 5.83±0.90(a,α) | 6.50±0.46(b,α) | 5.47±0.47(a,α) | 1.293 | 0.293 |
| BFL-15 | 7.63±1.27(b,α) | 5.67±1.04(bc,β) | 5.60±0.30(a,β) | 6.285 | 0.006 |
| F | 5.758 | 4.282 | 0.494 |  |  |
| P | 0.004 | 0.015 | 0.69 |  |  |

Table S3. Full two-way ANOVA output for flexural strength at 28 days.

|  | BFC-0.1 | BFC-0.3 | BFC-0.5 | F | P |
| --- | --- | --- | --- | --- | --- |
| BFL-6 | 6.47±0.55(a,α) | 6.17±0.71(ab,α) | 8.20±1.04(a,β) | 7.229 | 0.003 |
| BFL-9 | 6.80±0.46(a,αβ) | 6.30±0.69(ab,α) | 7.67±0.32(a,β) | 2.869 | 0.076 |
| BFL-12 | 7.10±0.85(a,α) | 6.90±0.52(a,α) | 6.57±0.74(b,α) | 0.436 | 0.652 |
| BFL-15 | 6.73±0.50(a,α) | 5.60±0.26(b,β) | 7.43±1.20(ab,α) | 5.136 | 0.014 |
| F | 0.406 | 1.708 | 2.778 |  |  |
| P | 0.75 | 0.192 | 0.063 |  |  |

Table S4. Full two-way ANOVA output for compressive strength at 3 days.

|  | BFC-0.1 | BFC-0.3 | BFC-0.5 | F | P |
| --- | --- | --- | --- | --- | --- |
| BFL-6 | 32.47±2.73(a,α) | 30.55±1.85(a,β) | 32.67±1.55(a,α) | 2.546 | 0.087 |
| BFL-9 | 29.32±1.77(b,α) | 29.08±0.73(a,α) | 31.83±1.25(a,β) | 4.334 | 0.017 |
| BFL-12 | 33.82±2.23(a,α) | 33.67±1.40(b,α) | 33.08±1.38(a,α) | 0.28 | 0.757 |
| BFL-15 | 29.68±2.62(b,α) | 30.35±1.67(a,αβ) | 31.62±1.28(a,β) | 1.798 | 0.174 |
| F | 8.848 | 7.07 | 0.89 |  |  |
| P | 0 | 0 | 0.451 |  |  |

Table S5. Full two-way ANOVA output for compressive strength at 7 days.

|  | BFC-0.1 | BFC-0.3 | BFC-0.5 | F | P |
| --- | --- | --- | --- | --- | --- |
| BFL-6 | 36.32±2.08(a,α) | 31.25±1.39(a,β) | 33.82±1.82(a,γ) | 10.583 | 0 |
| BFL-9 | 32.32±1.36(b,α) | 33.80±2.57(bc,α) | 33.75±1.76(a,α) | 1.17 | 0.317 |
| BFL-12 | 33.80±2.21(bc,α) | 35.53±0.98(c,αβ) | 35.83±2.41(b,β) | 1.987 | 0.146 |
| BFL-15 | 35.62±2.08(ac,α) | 32.82±1.17(ab,β) | 37.45±2.28(b,α) | 8.978 | 0 |
| F | 5.388 | 5.311 | 5.21 |  |  |
| P | 0.002 | 0.003 | 0.003 |  |  |

Table S6. Full two-way ANOVA output for compressive strength at 28 days.

|  | BFC-0.1 | BFC-0.3 | BFC-0.5 | F | P |
| --- | --- | --- | --- | --- | --- |
| BFL-6 | 34.63±2.55(a,α) | 34.78±3.45(ac,α) | 32.45±3.41(a,α) | 1.468 | 0.239 |
| BFL-9 | 35.65±1.47(a,αβ) | 33.02±3.07(a,α) | 35.9±3.14(bc,β) | 2.196 | 0.12 |
| BFL-12 | 38.95±1.72(b,α) | 38.70±2.95(b,α) | 37.10±2.31(bc,α) | 0.867 | 0.425 |
| BFL-15 | 35.20±1.58(a,α) | 35.70±2.13(ac,α) | 35.15±2.86(ac,α) | 0.08 | 0.924 |
| F | 3.237 | 4.862 | 3.343 |  |  |
| P | 0.028 | 0.004 | 0.025 |  |  |
